# The *Campylobacter jejuni* BumS sensor phosphatase detects the branched short-chain fatty acids isobutyrate and isovalerate as direct cues for signal transduction

**DOI:** 10.1128/mbio.03278-24

**Published:** 2024-12-13

**Authors:** Nestor Ruiz, Jiawei Xing, Igor B. Zhulin, Chad A. Brautigam, David R. Hendrixson

**Affiliations:** 1 Department of Microbiology, University of Texas Southwestern Medical Center, Dallas, Texas, USA; 2 Department of Microbiology, The Ohio State University, Columbus, Ohio, USA; 3 Translational Data Analytics Institute, The Ohio State University, Columbus, Ohio, USA; 4 Department of Biophysics, University of Texas Southwestern Medical Center, Dallas, Texas, USA; Fred Hutchinson Cancer Center, Seattle, Washington, USA

**Keywords:** *Campylobacter jejuni*, two-component signal transduction system, BumS, sensor phosphatase, isobutyrate, isovalerate, butyrate

## Abstract

Two-component signal transduction systems (TCSs) are nearly ubiquitous across bacterial species and enable bacteria to sense and respond to specific cues for environmental adaptation. The *Campylobacter jejuni* BumSR TCS is unusual in that the BumS sensor exclusively functions as a phosphatase rather than a kinase to control phosphorylated levels of its cognate BumR response regulator (P-BumR). We previously found that BumSR directs a response to the short-chain fatty acid butyrate generated by resident microbiota so that *C. jejuni* identifies ideal lower intestinal niches in avian and human hosts for colonization. However, butyrate is an indirect cue for BumS and did not inhibit *in vitro* BumS phosphatase activity for P-BumR. In this work, we expanded the repertoire of lower intestinal metabolites that are cues sensed by BumS that modulate the expression of genes required for colonization to include the branched short-chain fatty acids isobutyrate and isovalerate. Unlike butyrate, isobutyrate and isovalerate inhibited *in vitro* BumS phosphatase activity for P-BumR, indicating that these metabolites are direct cues for BumS. Isobutyrate and isovalerate reduced the thermostability of BumS and caused a reorganization of protein structure to suggest how sensing these cues inhibits phosphatase activity. We also identified residues in the BumS sensory domain required to detect isobutyrate, isovalerate, and butyrate and for optimal colonization of hosts to reveal how gut bacteria can recognize these intestinal metabolites. Our work reveals how this unusual bacterial sensor phosphatase senses a repertoire of intestinal metabolites and how cues alter BumSR signal transduction to influence *C. jejuni* colonization of hosts.

**IMPORTANCE:**

TCSs are prevalent in many bacteria, but the cues sensed by each are not actually known for many of these systems. Microbiota-generated butyrate in human and avian hosts is detected by the *Campylobacter jejuni* BumS sensor phosphatase so that the bacterium identifies ideal lower intestinal niches for colonization. However, BumS only indirectly senses butyrate to inhibit dephosphorylation of its cognate BumR response regulator. Here, we expanded the repertoire of cues sensed by BumS to the branched-short chain fatty acids isobutyrate and isovalerate that are also abundant in the lower intestines. Both isobutyrate and isovalerate are potent, direct cues for BumS, whereas butyrate is an indirect cue. Leveraging isobutyrate and isovalerate as direct cues, we reveal BumS structure is altered upon cue detection to inhibit its phosphatase activity. We provide an understanding of the mechanics of an unusual mode of signal transduction executed by BumSR and other bacterial sensor phosphatase-driven TCSs.

## INTRODUCTION

**Fig 1:**
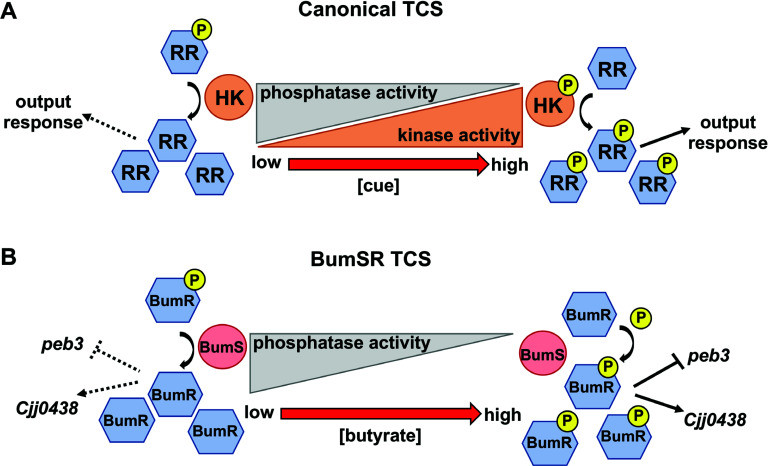
Comparative signal transduction mechanisms of canonical TCS and the sensor phosphatase-driven BumSR TCS. (**A**) Most TCSs use a cue-dependent sensor histidine kinase (HK) with an opposing phosphatase activity to control phosphorylated levels of a cognate response regulator (RR) for an output response. For example, the HK pool may shift toward a net kinase activity to increase the levels of P-RR to mediate a response as cue concentrations increase. (**B**) In contrast, *Campylobacter jejuni* BumS is a sensor phosphatase whose activity is modulated by cues to control the level of phosphorylated BumR, which must be modified with a non-cognate endogenous phosphodonor to alter the transcription of target genes. High P-BumR levels lead to increased DNA binding to cause transcriptional activation of some genes such as *Cjj0438* and transcriptional repression of other genes such as *peb3*. Decreased phosphorylation of BumR leads to reduced transcription of *Cjj0438* and increased derepression of transcription of *peb3*.

Two-component signal transduction systems (TCSs) are one of the most abundant signal transduction systems in nature, nearly ubiquitous in bacteria and present in many archaea and some fungi and plants (1, 2). Canonical TCSs employ a sensor histidine kinase (HK) to detect one or more specific exogenous or intracellular cues to influence its autophosphorylation on a conserved histidine residue (3–5) (Fig. 1A). Subsequent signal transduction occurs via autophosphorylation of the cognate response regulator (RR) using the phosphohistidine of the HK as a substrate (3, 4). Phosphorylation alters the ability of the RR to mediate a response. For many DNA-binding RRs, this modification can cause a change in dimerization and affinity for specific promoters to activate or repress transcription of specific genes (6, 7). Many TCS sensors also function as phosphatases when not active as autokinases to dephosphorylate their cognate RR (8, 9). This phosphatase activity lowers levels of the phosphorylated RR, which is important to reset the system in the absence of a cue, improve signaling fidelity, and prevent crosstalk of the RR with other TCSs or non-cognate phosphodonors in the bacterium (10). Despite the plethora of bacterial TCSs that have been discovered, the specific cues sensed by many HKs are unknown, and a greater understanding of how sensing cues impact the enzymatic activities of HKs for signal transduction is needed (11). Furthermore, the phosphatase activity of TCS HKs and how cues influence phosphatase activity are vastly underexplored compared with the kinase activity of these sensors.

We previously discovered that the *C. jejuni* BumSR TCS is required to sense physiological levels of the short-chain fatty acid (SCFA) butyrate (10–34 mM) generated by the microbiota in the lower intestinal tract of human and avian hosts (12–18). Upon sensing exogenous butyrate, BumSR altered transcription of over 14 genes (12, 13, 19). Transcription of some genes, such as *peb3,* was repressed in the presence of exogenous butyrate, whereas transcription of others like *Cjj81176_0438* and *Cjj81176_0439* was augmented (Fig. 1B). *peb3* encodes a putative transporter for phosphate-containing metabolites (20, 21). *Cjj81176_0438* (hereafter referred to as “*Cjj0438”* for simplicity), with the downstream gene *Cjj81176_0439,* encodes a gluconate dehydrogenase complex (22). Both *peb3* and *Cjj0438*, along with many other genes within the BumSR regulon, are required by *C. jejuni* for optimal levels of commensal colonization of the chick’s lower intestinal tract (12, 22). Because butyrate is abundant in the lower intestinal tract of hosts, which is the favored niche colonized by *C. jejuni*, we proposed that sensing butyrate via the BumSR TCS allows *C. jejuni* to discern between intestinal regions to specifically identify lower intestinal niches ideal for infection of humans to initiate diarrheal disease and avian hosts to promote commensalism (12). As such, *C. jejuni* Δ*bumR* was less fit for colonization of the lower intestinal tract of chickens and essential for infection of human volunteers to lead to diarrheal disease (12, 19, 23). *C. jejuni* Δ*bumS* showed very subtle differences in colonization of the chick intestinal tract compared with wild-type (WT) *C. jejuni* (12).

In contrast to many canonical TCSs in which the sensor possesses both kinase and phosphatase activity to influence the level of phosphorylation of its cognate RR to mediate a response (Fig. 1A), we discovered that the BumS sensor of the *C. jejuni* BumSR TCS had no discernable *in vitro* autokinase activity (12). Instead, BumS exclusively functioned as a sensor phosphatase to control the level of phosphorylation of its cognate RR, BumR. (Fig. 1B). Unlike many HKs, bioinformatic analysis suggested that BumS is a cytoplasmic sensor and lacks conserved residues usually required for HK autophosphorylation (12). The increase in transcriptional repression or activation of specific genes upon exposure of *C. jejuni* to butyrate is consistent with the inhibition of BumS phosphatase activity to augment cellular P-BumR levels, with P-BumR possessing a higher affinity for its target promoters compared with unphosphorylated BumR to serve as both a transcriptional repressor or activator, depending on the promoter (12). However, butyrate did not inhibit the *in vitro* phosphatase activity of recombinant BumS for P-BumR (12). Thus, we proposed that BumS indirectly senses butyrate as a cue. We hypothesize that butyrate may be converted into a cue upon entry into *C. jejuni* or may stimulate the production of a cue by *C. jejuni* directly sensed by BumS to promote signal transduction and alter gene transcription through BumR. Since our discovery of BumSR as the first known bacterial TCS to exclusively employ a sensor phosphatase-driven signal transduction mechanism, other sensor phosphatase-driven TCSs have been identified (24–26), suggesting that these TCSs represent a growing family of bacterial TCSs.

In this work, we expanded the repertoire of cues sensed by the BumSR TCS by identifying the branched short-chain fatty acids (BSCFAs) isobutyrate and isovalerate as direct cues detected by the BumS sensor phosphatase. These intestinal metabolites are generated by the gut microbiota of the lower intestinal tract during fermentation of branched-chain amino acids (18, 27–31). The BumSR TCS sensed and responded to these cues at their physiological concentration in the lower intestines of human and avian hosts where *C. jejuni* resides. In contrast to butyrate, isobutyrate and isovalerate inhibited BumS dephosphorylation of P-BumR, indicating that the BSCFAs are directly sensed by BumS. We also identified residues in the sensing domain of BumS required to sense butyrate, isobutyrate, and isovalerate. Additionally, a BumS mutant that was partially blind to these cues was severely attenuated for colonization throughout the chick intestinal tract. Our findings expand our understanding of how a TCS of an intestinal bacterium recognizes and senses these SCFA and BSCFA intestinal metabolites and the mechanics of an unusual sensor phosphatase-driven TCS to mediate signal transduction and alter a transcriptional response required for infection of hosts.

## RESULTS

### Isobutyrate and isovalerate are direct cues for BumS that inhibit its phosphatase activity

We previously discovered that the BumS phosphatase mediated response to exogenous butyrate at physiological levels found in the lower intestinal regions of chickens and humans (10–34 mM [14–18]). Despite exposure to exogenous butyrate causing a BumSR-dependent response, *in vitro* analysis of purified BumS revealed that BumS phosphatase activity was not inhibited by physiological concentrations of butyrate, suggesting that butyrate likely functions as an indirect cue for BumS in *C. jejuni*. We queried whether BumS has an expanded cue repertoire to directly or indirectly sense other relevant intestinal metabolites. We postulated that a cue sensed by BumS may be structurally similar to butyrate and abundant in the lower intestinal tract of both the human and avian hosts where *C. jejuni* normally resides. We analyzed a panel of BSCFAs and branched-chain α-ketoacid metabolites that met these requirements. The BSCFAs isobutyrate and isovalerate are end products of the fermentation of valine and leucine, respectively, by the gut microbiota (27, 32). Both isobutyrate and isovalerate have been reported at levels up to 3 mM in the lower intestinal tract of humans and chickens (18, 27–31). The branched-chain α-ketoacids 4-methyl-2-oxovalerate, 3-methyl-2-oxobutyrate, and 3-methyl-2-oxopentanoate are intermediates produced during the fermentation of leucine, valine, and isoleucine, respectively, for the production of BSCFAs (27, 32).

**Fig 2:**
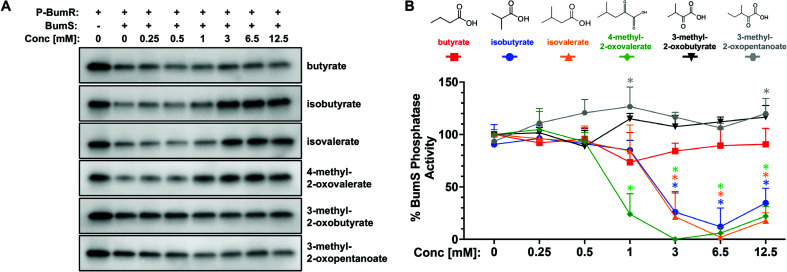
BumS phosphatase activity for P-BumR with and without potential intestinal metabolic cues. (**A**) Dephosphorylation of ^32^P-BumR to BumR by BumS in the presence of metabolites shown in panel **B**. Indicated concentrations of metabolites were added to BumS before addition to ^32^P-BumR. (**B**) Quantitation of the level of BumS phosphatase activity by densitometry. Assays were performed in triplicate with one assay shown in panel **A**. The level of ^32^P-BumR remaining at the end of the assay for each reaction was compared with ^32^P-BumR alone. Percent BumS phosphatase activity was calculated relative to that of WT BumS without metabolites, which was set at 100%. Points indicate the mean BumS phosphatase activity with each metabolite at each concentration. Error bars indicate standard deviation. Statistical significance of the difference in BumS phosphatase activity with the metabolite at the indicated concentration compared with BumS without the metabolite was calculated by analysis of variance (ANOVA) multiple comparison test (*, *P* < 0.05 between the metabolite at the indicated concentration).

To assess whether these metabolites are direct cues sensed by BumS, we compared the level of BumS phosphatase activity for P-BumR in the presence and absence of the metabolites (Fig. 2A and B). As previously reported (12), BumS dephosphorylated P-BumR in the absence of cues (Fig. 2A, lane 2 of panels), and its phosphatase activity was not inhibited by up to 12.5 mM butyrate (Fig. 2A, top row). However, BumS phosphatase activity was gradually inhibited upon incubation with >1 mM isobutyrate, isovalerate, and 4-methyl-2-oxovalerate to retain high levels of P-BumR (Fig. 2A). Quantitation of the final P-BumR levels in the reactions by densitometry confirmed that these metabolites inhibited greater than 70% of the phosphatase activity of BumS (Fig. 2B). In contrast, two branched-chain α-ketoacids, 3-methyl-2-oxobutyrate, and 3-methyl-2-oxopentanoate did not inhibit the phosphatase activity of BumS (Fig. 2A and B). These findings indicated that *in vitro* BumS recognizes specific BSCFAs and 4-methyl-2-oxovalerate as direct cues. However, the concentration of 4-methyl-2-oxovalerate that inhibited BumS phosphatase activity (> 1 mM) is orders of magnitude greater than expected in the host lower intestinal tract or in *C. jejuni* as an intermediate metabolite during leucine catabolism (33). Thus, this metabolite is likely not a biologically relevant cue. Based on these *in vitro* phosphatase assays, we identified the BSCFAs isobutyrate and isovalerate as direct intestinal metabolite cues for BumS. Therefore, we focused our analysis on the ability of the BumSR TCS to sense and respond to isobutyrate, isovalerate, and butyrate for the remainder of this study.

### Sensing exogenous BSCFAs through the *C. jejuni* BumSR TCS

**Fig 3:**
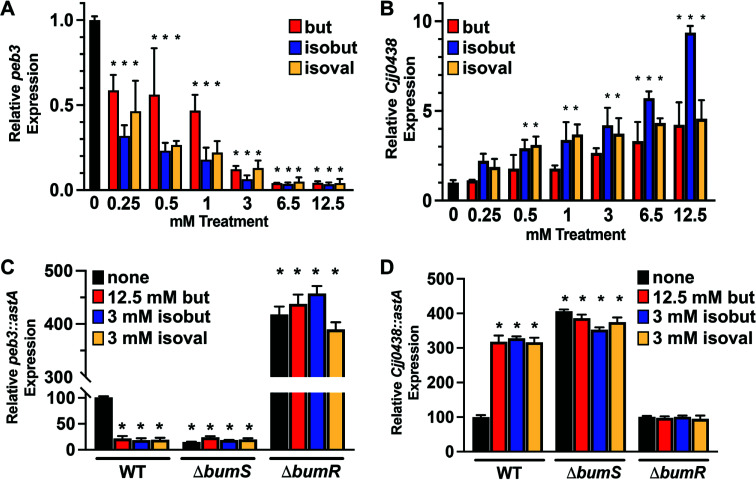
Expression of *peb3* and *Cjj0438* in *C. jejuni* grown with metabolites. (A and B) qRT-PCR analysis of (**A**) *peb3* and (**B**) *Cjj0438* transcription in WT *C. jejuni* grown in CDM (black) or CDM with butyrate (red), isobutyrate (blue), or isovalerate (gold) at the indicated concentration. Level of expression in WT with metabolites is relative to WT in CDM alone, which was set to 1. The results from a representative assay with WT *C. jejuni* tested with each metabolite in triplicate are shown. Error bars indicate standard deviations of the average level of gene expression. Statistical significance in *peb3* or *Cjj0438* expression with the inclusion of a metabolite at the indicated concentration compared with the absence of any metabolite was calculated by ANOVA with Tukey’s test (*, *P* < 0.05 between the metabolite at the indicated concentration). (C and D) Expression of (**C**) *peb3::astA* and (**D**) *Cjj0438::astA* in WT *C. jejuni*, Δ*bumS*, or Δ*bumR* grown in MH media alone (black) or with 12.5 mM butyrate (red), 3 mM isobutyrate (blue), or 3 mM isovalerate (gold). Reporter activity was monitored by arylsulfatase assays. Expression level in each strain is relative to WT in MH media alone, which was set to 100 units. The results from a representative assay with strains tested with each metabolite in triplicate are shown. Error bars indicate standard deviations of the average level of gene expression. Statistical significance in *peb3::astA* or *Cjj0438::astA* expression in the presence of a metabolite at the indicated concentration compared with the absence of any metabolite was calculated by ANOVA with Tukey’s test (*, *P* < 0.05 between the metabolite at the indicated concentration).

We assessed whether *C. jejuni* sensed exogenous isobutyrate and isovalerate at physiological levels like butyrate to affect signal transduction and expression of the BumSR regulon. Although butyrate has been reported up to 30 mM in the human and avian lower intestinal tract, isobutyrate and isovalerate have been found between 0.25 and 3 mM (18, 27–31). We first grew *C. jejuni* in a *Campylobacter*-defined medium (CDM), which contains all 20 amino acids and specific keto acids as primary carbon sources, with increasing concentrations of 0.25–12.5 mM butyrate, isobutyrate, or isovalerate and then compared the level of *peb3* or *Cjj0438* expression via qRT-PCR. As previously reported, increasing concentrations of exogenous butyrate caused a 2-fold to 25-fold repression of *peb3* transcription ([Fig. 3A]; [12]). Increasing concentrations of both isobutyrate and isovalerate similarly repressed *peb3* expression (Fig. 3A). We observed a greater repression of *peb3* expression with isobutyrate and isovalerate than butyrate, especially between 0.5 and 1 mM, which is within the physiological range of these metabolites. For *Cjj0438* expression, all metabolites positively influenced expression with a 4-fold or greater expression at the highest concentration of metabolites tested (12.5 mM) (Fig. 3B). We observed a greater increase in *Cjj0438* expression with 0.5–3 mM isobutyrate or isovalerate than we did with the same concentrations of butyrate. Although we observed a 10-fold increase in *Cjj0438* expression with isobutyrate up to 12.5 mM, this concentration is outside its physiological range in the lower intestinal tract of hosts. These results suggest that *C. jejuni* is more sensitive to lower concentrations of isobutyrate and isovalerate than butyrate, presumably detected by BumS.

We next validated that the BumSR TCS in *C. jejuni* directed a response to exogenous isobutyrate and isovalerate to alter expression of the BumSR regulon. We compared expression of *peb3::astA* and *Cjj0438::astA* transcriptional reporters on the chromosome of WT *C. jejuni* and Δ*bumS* or Δ*bumR* mutants after growth in Mueller-Hinton (MH) media with 12.5 mM butyrate, 3 mM isobutyrate, or 3 mM isovalerate, which are physiologically relevant levels of the metabolites in the intestinal tract of hosts. Although we previously monitored expression of the BumSR regulon in CDM, we used MH media with defined concentrations of the metabolites to efficiently execute all subsequent experiments. We observed identical trends for WT *C. jejuni* in alteration of gene expression in MH with metabolites as we did with CDM with metabolites (Fig. 3A through D). When WT *C. jejuni* was grown with any cue, *peb3::astA* expression was repressed 5-fold relative to the absence of metabolites (Fig. 3C). In Δ*bumS* without metabolites*, peb3::astA* expression was repressed compared with WT, which is consistent with the accumulation of P-BumR due to the lack of BumS phosphatase activity. The lack of BumR in Δ*bumR* fully derepressed *peb3::astA* expression. For both Δ*bumS* and Δ*bumR* mutants, the presence of metabolites in the media did not alter *peb3::astA* expression. Consistent with CDM-grown WT *C. jejuni, Cjj0438::astA* expression was enhanced in WT *C. jejuni* grown in MH with any of the three metabolites (Fig. 3D). Similar to *peb3::astA*, we observed no change in the expression of *Cjj0438::astA* when Δ*bumS* or Δ*bumR* were grown in the presence of any metabolite (Fig. 3D). These data indicated that the BumSR TCS is required for *C. jejuni* to sense exogenous isobutyrate and isovalerate and respond to these metabolites to alter expression of the BumSR regulon.

### The BumSR TCS senses and responds to a complex mixture of metabolites

**Fig 4:**
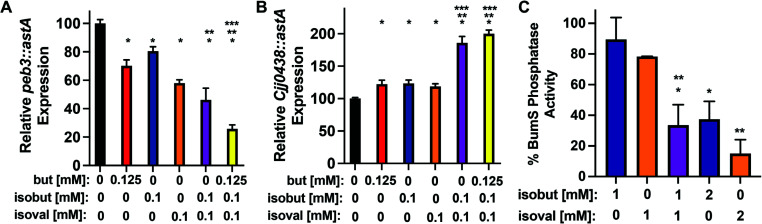
Analysis of the ability of the BumSR TCS to sense and respond to a metabolite mixture. (A and B) Expression of (**A**) *peb3::astA* and (**B**) *Cjj0438::astA* in WT *C. jejuni* grown in MH media alone (black) or with single metabolites or a mixture of two or more metabolites at the indicated concentrations. Reporter activity was monitored by arylsulfatase assays. Expression level in each strain is relative to WT in MH media alone, which was set to 100 units. The results from a representative assay with strains tested with each metabolite in triplicate are shown. Error bars indicate standard deviations of the average level of gene expression. Statistical significance in *peb3::astA* or *Cjj0438::astA* expression with inclusion of one or more metabolites at the indicated concentration was calculated by ANOVA with Tukey’s test (*, *P* < 0.05 between metabolites at the indicated concentration compared with the absence of any metabolite; **, *P* < 0.05 between a mixture of the indicated metabolites compared with isobutyrate alone; ***, *P* < 0.05 between a mixture of indicated metabolites compared with isovalerate alone). (**C**) Quantitation of the level of BumS phosphatase activity for ^32^P-BumR in the presence of isobutyrate and/or isovalerate at the indicated concentration. The level of ^32^P-BumR remaining at the end of the assay for each reaction was compared with ^32^P-BumR with WT BumS, which was set at 100%. Points indicate the mean phosphatase activity with each metabolite at each concentration assayed in triplicate. Error bars indicate standard deviation of the average BumS phosphatase activity. Statistical significance of difference in BumS phosphatase activity with the metabolites at the indicated concentration compared with BumS in the absence of the metabolite was calculated by ANOVA multiple comparison test (*, *P* < 0.05 between BumS with the metabolites at the indicated concentration compared with BumS with 1 mM isobutyrate; **, *P* < 0.05 between BumS the metabolites at the indicated concentration compared with BumS with 1 mM isovalerate).

The BumSR TCS responded to exogenous butyrate, isobutyrate, or isovalerate when they were added as the sole metabolites to the media. However, *C. jejuni* is presumably exposed to a complex mixture of these cues normally produced by the resident microbiota in its natural intestinal niche in hosts. Thus, we compared how BumSR TCS senses and responds to a mixture of subphysiological concentrations of each cue relative to a single metabolite. After growth in MH with 0.1 mM isobutyrate, 0.1 mM isovalerate, or 0.125 mM butyrate (approximately ~30 to 100 times lower than peak physiological levels of any one cue), *peb3::astA* expression was reduced 20%–42% in comparison to cells grown without metabolites (Fig. 4A). However, the presence of isobutyrate and isovalerate at 0.1 mM together repressed expression by 54%. Addition of 0.125 mM butyrate further reduced the expression to 75%. As shown in Fig. 2A, approximately 75% repression of *peb3* expression required 0.5 mM isobutyrate or isovalerate or 1–3 mM butyrate alone, which are higher molar concentrations than the 0.325 mM concentration of metabolites in the mixture added to the media. We observed similar trends for the expression of *Cjj0438*. Expression of *Cjj0438::astA* was only enhanced 20% by 0.1 mM isobutyrate or isovalerate (Fig. 4B). However, combining isobutyrate and isovalerate stimulated *Cjj0438::astA* expression by 80% and inclusion of butyrate increased the expression 2-fold (Fig. 4B). As shown in Fig. 2B, 2.5-fold to 4-fold higher levels of each single metabolite was required for a similar increase in *Cjj0438* expression compared with the concentration of each metabolite in a mixture. These observations support a trend in that these metabolites are potent cues for the BumSR TCS when present even at very low physiological concentrations in a mixture.

We next analyzed how a mixture of isobutyrate and isovalerate influenced the *in vitro* phosphatase activity of BumS. The presence of 1 mM isobutyrate or isovalerate did not significantly inhibit BumS phosphatase activity (Fig. 4C; Fig. S1). However, when combined at 1 mM final concentration for each, BumS phosphatase activity for P-BumR was inhibited by 66%. This level of inhibition was similar to the level of inhibition by 2 mM isobutyrate or 2 mM isovalerate alone (Fig. 4C; Fig. S1). In summary, BumS phosphatase activity is sensitive to inhibition upon exposure to a mixture of cues at low concentrations as it is with exposure to a single metabolic cue.

### Impact of BSCFA direct cues on BumS thermostability

**Fig 5:**
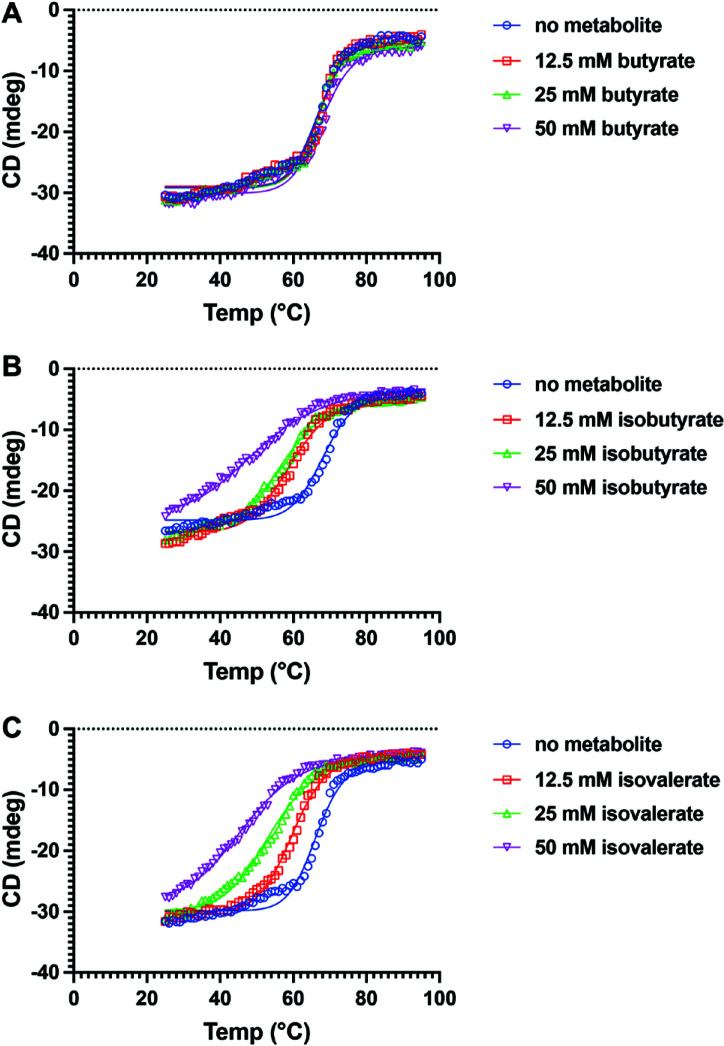
CD spectroscopy analysis of WT BumS with and without cues. Ellipticity (in machine units, i.e., millidegrees; mdeg) of purified WT BumS without metabolites or with (**A**) butyrate, (**B**) isobutyrate, or (**C**) isovalerate was measured at 222 nm. For (**A–C**), WT BumS was mixed without metabolites (blue, circles) or with 12.5 mM (red, squares), 25 mM (green, triangles), or 50 mM (purple, inverted triangles) of each metabolite. Lines represent fits of data using equation (1) (see Materials and Methods).

Our data suggested that the BSCFAs isobutyrate and isovalerate are sensed directly by BumS to inhibit its phosphatase activity for P-BumR. We investigated how these cues may impact BumS to alter its phosphatase activity. For this approach, we analyzed the circular dichroism of recombinant BumS at 222 nm with and without metabolites with increasing temperature. BumS alone and BumS with up to 50 mM butyrate displayed similar melt curves, suggesting a lack of direct interaction between BumS and butyrate (Fig. 5A). These data are consistent with our findings and hypothesis for butyrate as an indirect cue sensed by BumS. In contrast, we observed increasingly significant changes to the melt curve of BumS in the presence of isobutyrate and isovalerate as the concentrations of these metabolites increased from 12.5 to 50 mM (Fig. 5B and C). Specifically, isobutyrate and isovalerate had a net destabilizing effect on the thermostability of BumS. These data suggested that upon BumS binding isobutyrate and isovalerate, a reorganization of the tertiary structure of the protein may occur, rendering it less thermostable. These findings are consistent with the decrease in phosphatase activity we observed with BumS in the presence of increasing concentrations of isobutyrate or isovalerate.

### BumS detects BSCFAs and butyrate through specific residues of its PAS module

**Fig 6:**
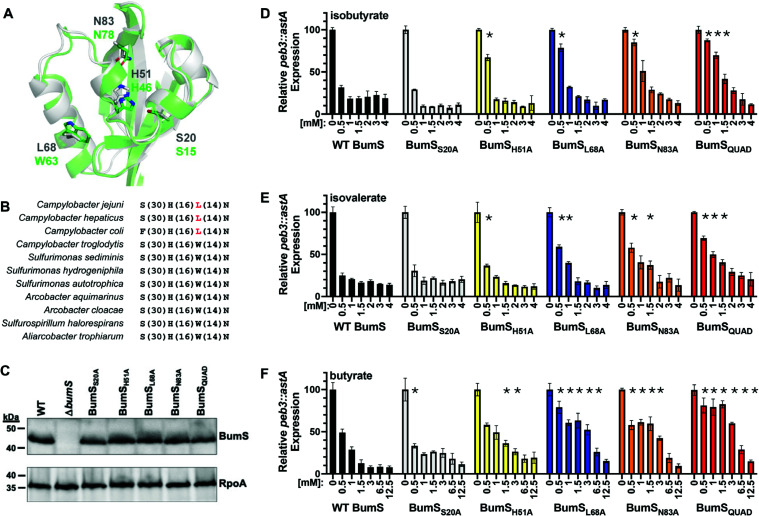
Binding sites in the BumS PAS domain. (**A**) Structural comparison of BumS and CetB PAS domains shown in gray and green, respectively. Key residues for ligand/cofactor binding are labeled on the structure. CetB PAS domain has a conserved tryptophan for FAD binding, whereas BumS PAS domain has a leucine substitution in this position. (**B**) Sequence conservation of selected homologous PAS domains from BumS orthologs and proteins from other *Campylobacterales* species (NCBI accessions: WP_153887626.1, WP_131952296.1, WP_148563445.1, WP_142692976.1, WP_193151068.1, WP_151901227.1, WP_013326056.1, WP_129094579.1, WP_129012495.1, WP_069478740.1, and WP_115428278.1). Only key residues are shown. Residue numbers between key residues are shown in parentheses. Substitutions from tryptophan to leucine are highlighted in red. (**C**). Immunoblot analysis of BumS in whole-cell lysates of WT *C. jejuni* or *C. jejuni* expressing indicated BumS mutants from the native chromosomal locus. Specific antiserum to BumS was used. Detection of RpoA serves as a control to ensure equal loading of proteins across strains. (**D–F**) Expression of *peb3::astA* in WT *C. jejuni* (black bars), *bumS*_S20A_ (grey bars), *bumS*_H51A_ (yellow bars), *bumS*_L68A_ (blue bars), *bumS*_N83A_ (orange bars), or *bumS*_QUAD_ (red bars) grown in the presence of the indicated concentrations of (**D**) isobutyrate, (**E**) isovalerate, or (**F**) butyrate. Transcriptional reporter activity was monitored by arylsulfatase assays. The level of expression in each strain with a metabolite is relative to the same strain grown without the metabolite, which was set to 100 units. The results from a representative assay with strains tested with each metabolite in triplicate are shown. Error bars indicate standard deviations of the average level of gene expression. Statistical significance in *peb3::astA* expression in each mutant strain with a particular concentration of metabolite compared with WT *C. jejuni* with the same concentration of metabolite was calculated by ANOVA with Tukey’s test (*, *P* < 0.05).

BumS is a cytoplasmic sensor phosphatase with the initial N-terminal 120 residues predicted to contain a PAS domain. We hypothesize the BumS PAS domain is required to sense isobutyrate and isovalerate directly and butyrate indirectly for signal transduction to impact expression of the BumSR regulon. We previously classified PAS domains into clusters based on sequence conservations and sensory functions (34). Based on this classification, the BumS PAS domain belongs to cluster 14 (Table S1), which includes well-characterized PAS domains that bind flavin adenine dinucleotide (FAD) as a cofactor, such as the Aer PAS domain from *E. coli* (35) and the CetB PAS domain from *C. jejuni* (36). We previously identified key residues for FAD-binding in these PAS domains (34, 37), including the invariable tryptophan at the long α-helix (Fig. 6A). However, in the BumS PAS domain, tryptophan in this position is substituted with leucine (L68; Fig. 6A), which should prevent FAD binding, as seen in the *E. coli* Aer mutant with a similar substitution (38). In comparing the ~500 PAS domains that are most similar to that of BumS in cluster 14, we found that nearly half contain leucine in the position usually occupied by tryptophan (Fig. 6B; Fig. S2). For BumS orthologs, this substitution is only present in PAS domain of BumS from *C. jejuni*, *Campylobacter hepaticus*, and *Campylobacter coli*, suggesting that this evolutionary event has occurred recently in the *Campylobacter* genus (Fig. 6B, highlighted in red). Notably, two other key residues (H51 and N83) remain fully conserved in BumS and its homologs with S20 highly conserved, and these residues may contribute to their potential sensory functions (Fig. 6B; Fig. S2).

To determine whether these conserved BumS PAS domain residues are involved in sensing one or more of its cues, we generated BumS variants in which each amino acid was altered alone (BumS_S20A_, BumS_H51A_, BumS_L68A_, or BumS_N83A_) or all four resides were altered together (BumS_QUAD_). These alterations did not impair the stability or levels of BumS in whole-cell lysates of *C. jejuni* (Fig. 6C). To assess the impact of each alteration on the ability of BumS to sense a particular cue, we grew each BumS variant in MH media in the presence of increasing concentrations of each metabolite and monitored the level of *peb3::astA* expression, which is normally repressed by the BumSR TCS in the presence of cues. WT BumS sensed increasing concentrations of isobutyrate from 0.5 to 4 mM to repress *peb3::astA* expression 69%–82% (Fig. 6D). We did not detect any differences in the ability of BumS_S20A_ to sense and respond to these levels of butyrate and repress *peb3::astA* expression, suggesting that S20 is not involved in sensing this metabolite. In contrast, we observed a reduced ability of BumS_H51A_, BumS_L68A_, and BumS_N83A_ to sense isobutyrate at the lowest concentration (0.5 mM) and fully repress *peb3::astA* expression (Fig. 6D). These alterations did not significantly impact the ability of BumS to sense isobutyrate at higher concentrations. However, *C. jejuni* producing BumS_QUAD_ with all of the alterations was more defective in sensing isobutyrate up to 1.5 mM to fully repress *peb3::astA* expression in comparison to WT BumS (Fig. 6D). At the highest levels of isobutyrate, BumS_QUAD_ sensed isobutyrate to repress *peb3::astA* expression similar to WT (Fig. 6D).

For isovalerate, we observed a similar trend of the variants in their ability to sense and respond to this metabolite (Fig. 6E). BumS_S20A_ showed no defects in sensing and responding to any concentration of isovalerate, and BumS_H51A_ only demonstrated a mild defect in sensing isovalerate at 0.5 mM. We observed that BumS_L68A_ and BumS_N83A_ demonstrated a modestly greater defect in sensing and responding to isovalerate than isobutyrate up to 1–1.5 mM. Like for isobutyrate, BumS_QUAD_ demonstrated a defect in sensing isovalerate up to 1.5 mM compared with WT BumS (Fig. 6E).

When we analyzed the ability of these BumS variants to sense and respond to butyrate, BumS_S20A_ was not defective for sensing butyrate. Additionally, we observed a mild, but inconsistent, impairment of BumS_H51A_ to sense butyrate and repress *peb3::astA* expression (Fig. 6F). However, alteration of L68 and N83 alone impacted the ability of BumS to sense and respond to butyrate up to 3–6.25 mM. As with isobutyrate and isovalerate, BumS_QUAD_ had the greatest defects in sensing and responding to butyrate, with significant differences compared with WT BumS in the ability to sense this metabolite at all concentrations tested (Fig. 6F). Since the alteration of L68 and N83 significantly contributed to the ability of BumS to sense and respond to isovalerate, isobutyrate, and butyrate, we conclude that detection of all of cues either by direct or indirect means requires at least these two specific residues.

**Fig 7:**
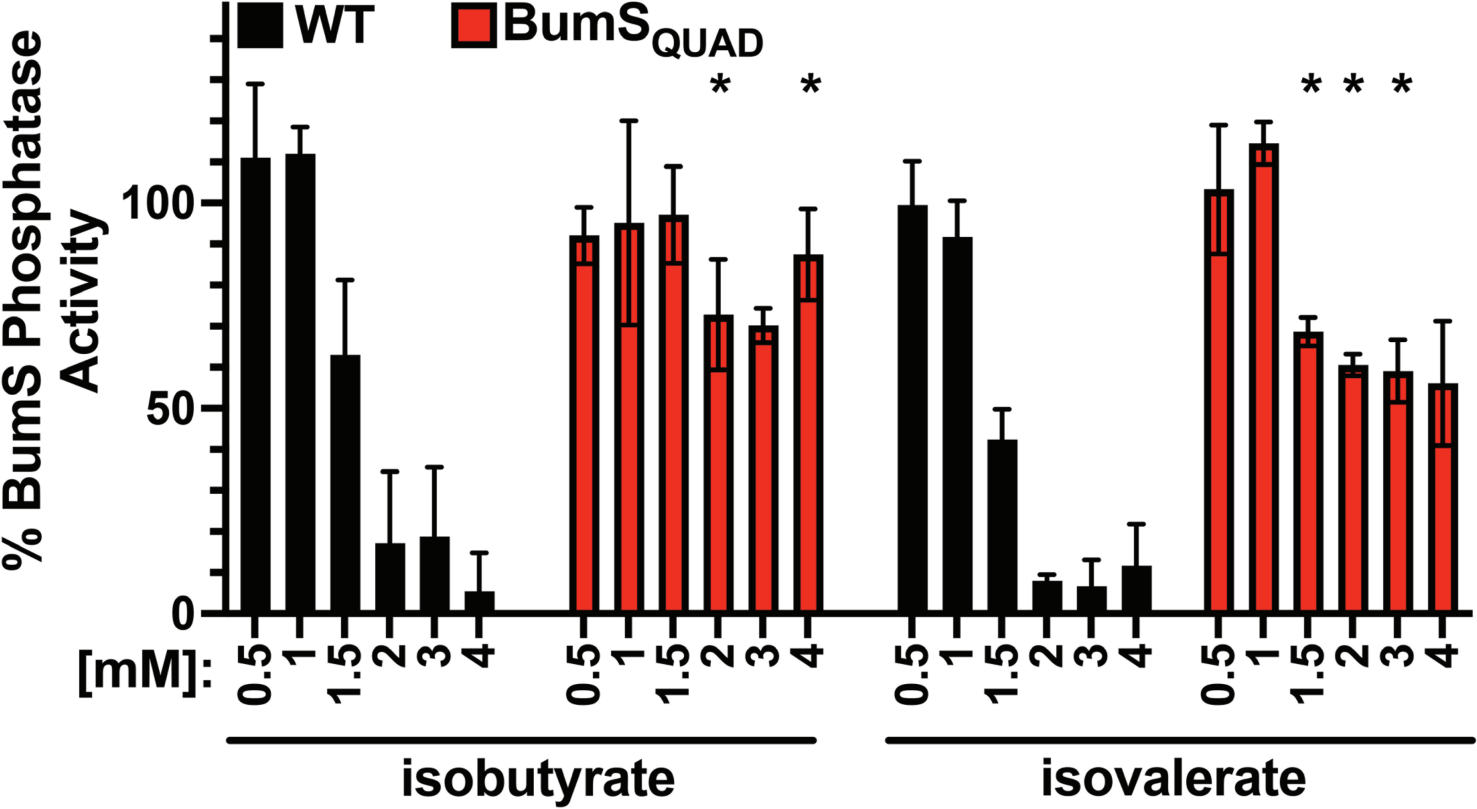
Effect of BumS PAS domain mutations on sensing cues for regulation of gene expression. Quantitation of the level of WT BumS or BumS_QUAD_ phosphatase activity for P-BumR in the presence of isobutyrate or isovalerate at the indicated concentration. Percent phosphatase activity for WT BumS or BumS_QUAD_ with metabolites was calculated based on the level of ^32^P-BumR remaining at the end of the assay relative to each protein without metabolites. Error bars indicate standard deviation of the average BumS phosphatase activity from three samples. Statistical significance of the difference in BumS_QUAD_ phosphatase activity with the metabolites at the indicated concentration compared with WT BumS with the same level of metabolite was calculated by ANOVA multiple comparison test (*, *P* < 0.05).

Of all the BumS point mutants analyzed, BumS_QUAD_ was the most impaired for sensing cues in *C. jejuni* to affect repression of *peb3* expression. Therefore, we characterized whether BumS_QUAD_ was defective in directly sensing isovalerate and isobutyrate *in vitro* to impact its phosphatase activity. Recombinant BumS_QUAD_ was purified and incubated with up to 4 mM isobutyrate or isovalerate and then assessed for dephosphorylation of P-BumR (Fig. 7; Fig. S3). Concentrations of 1.5–4 mM isobutyrate or isovalerate inhibited 40%–95% of the WT BumS phosphatase activity (Fig. 7; Fig. S3). However, at these concentrations of isobutyrate or isovalerate, BumS_QUAD_ phosphatase activity remained relatively high in all conditions when compared with WT BumS and was never inhibited more than ~44% by these metabolites. Therefore, *in vitro* BumS_QUAD_ was defective in the detection of isobutyrate or isovalerate as direct cues. Based on our gene expression and phosphatase assays, our data suggested that residues such as L68 and N83, and presumably others that remain to be identified, facilitate BumS to detect these intestinal metabolites for *C. jejuni*.

### A reduced ability of BumS to sense cues attenuates commensal colonization of the avian host

**Fig 8:**
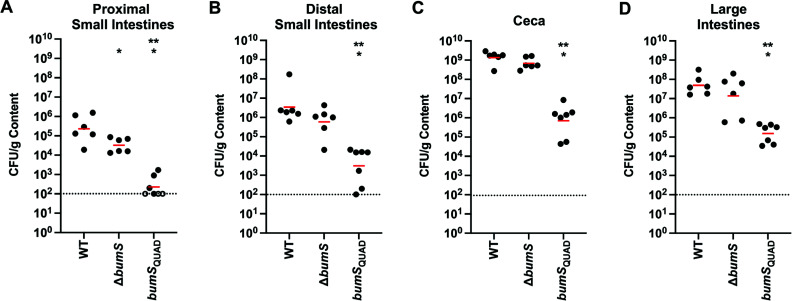
Colonization dynamics of WT *C. jejuni* and isogenic Δ*bumS* and *bumS*_QUAD_ mutants in the avian intestinal tract. Day of hatch chicks were orally infected with approximately 10–200 CFU of WT *C. jejuni*, isogenic Δ*bumS*, and *bumS*_QUAD_ mutants. Chicks were sacrificed at day 7 post-infection, and the levels of each *C. jejuni* strain in (**A**) the proximal small intestines, (**B**) distal small intestines, (**C**) ceca, and (**D**) large intestines were determined (reported as CFU per gram of content). Each closed circle represents the level of *C. jejuni* in a single chick. Open circles represent chicks with *C. jejuni* levels below the limit of detection (<100 CFU per gram of content; dotted lines). Red bars represent the geometric mean for each group. Statistical analysis was performed using the Mann-Whitney *U* test (*, *P* < 0.05 between Δ*bumS* or *bumS*_QUAD_ mutants and WT *C. jejuni*; **, *P* < 0.05 between *bumS*_QUAD_ and Δ*bumS* or mutants).

Since BumS_QUAD_ is partially blind to its cues *in vitro*, we assessed whether the *C. jejuni bumS*_QUAD_ mutant may be hindered *in vivo* for the detection of BSCFA and SCFA cues and attenuated for commensal colonization of the avian intestinal tract. Day-of-hatch chicks were orally inoculated with ~10–200 cfu of WT *C. jejuni*, the Δ*bumS* mutant, or *bumS*_QUAD_ mutant. At day 7 post-infection, the levels of cfu were enumerated in the upper portion of the small intestines (proximal small intestines), the lower portion of the small intestines (distal small intestines), ceca, and large intestines. Similar to our previous findings, we found that all strains showed the highest level of colonization in the ceca and large intestines, which are the preferred niches for *C. jejuni*, compared with the small intestinal regions (Fig. 8; [12, 39]). Like before (12), we found very little differences in the colonization capacities of WT *C. jejuni* and Δ*bumS*. The Δ*bumS* mutant generally colonized the avian intestinal tract 2-fold to 7-fold lower than WT, with the only significant difference in colonization occurring in the proximal small intestines (Fig. 8). These results imply that the lack of BumS, which would cause high levels of P-BumR and high levels of transcriptional activation of some genes such as *Cjj0438* and transcriptional repression of others like *peb3*, does not have a large impact on the short-term colonization of *C. jejuni* for the chick intestinal tract. In contrast, *bumS*_QUAD_ displayed a greatly reduced ability to colonize all areas of the chick intestinal tract. Compared with the WT *C. jejuni*, *bumS*_QUAD_ colonized at levels 1025-fold to 1928-fold lower in the small intestines and the ceca, and 321-fold lower in the large intestines (Fig. 8). Compared with *C. jejuni* Δ*bumS*, the *bumS*_QUAD_ mutant colonized all areas of the intestinal tract at lower levels, with 91-fold to 190-fold reductions in the small and large intestines, and 953-fold lower levels in the ceca. These results suggest that reducing the ability of BumS to sense its cues impacts its ability to properly alter P-BumR levels in the presence of these cues *in vivo*, resulting in dysregulation of expression of various genes in the BumSR regulon and hindering the ability of *C. jejuni* to colonize its natural host.

## DISCUSSION

Monitoring environments and intracellular homeostasis are vital for bacteria to respond to changing conditions and flourish in their respective niches. TCSs are present in nearly all bacterial species, with pathogenic and host-associated bacteria employing these systems to detect specific cues and alter gene expression for fitness and virulence processes. Numerous bacterial TCSs have been identified and analyzed, but for many, the cues specifically detected by each are not known. An understanding of how detection of cues mechanistically impacts enzymatic activities of the sensor to alter the levels of phosphorylation of the cognate RR to mediate a response is also lacking for most TCSs.

Prior to this study, we identified the SCFA butyrate as a cue for the *C. jejuni* BumSR TCS to influence transcriptional repression and activation of specific genes required for colonization (12, 13). In this study, we expanded the repertoire of cues sensed by the cytoplasmic BumS sensor to the BSCFAs isobutyrate and isovalerate. Butyrate, isobutyrate, and isovalerate are metabolites specifically enriched in the lower intestinal tract of avian species and humans, which are optimal niches colonized by *C. jejuni* in these hosts (14–18, 40–45). These metabolites are generated by the gut microbiota during the fermentation of specific branched-chain amino acids and carbohydrates. Because the BumSR TCS is required for WT levels of commensal colonization of chickens and infection of humans for diarrheal disease (12, 23), we postulate that BumSR specifically senses isobutyrate, isovalerate, and butyrate individually and collectively to identify lower intestinal niches for colonization and virulence.

We found that each single cue was detected *in vitro* at its *in vivo* physiological concentration by *C. jejuni* via the BumSR TCS to modulate expression of the BumSR regulon. However, the cues together at subphysiological concentrations stimulated the BumSR TCS as well as, if not better than, each cue alone at higher concentrations. The concentrations of these metabolites likely are in flux in the lower intestines due to the changing diet or metabolism of the host. Thus, the BumSR TCS may have advantageously evolved to detect multiple cues as specific landmarks of the lower intestinal tract in hosts. Detecting more than one cue solely or multiple cues together at lower concentration imparts *C. jejuni* with an enhanced ability to discern between different intestinal regions and identify ideal lower intestinal sites for infection. Having specific cues that are products of metabolism by the gut microbiota presents another advantage afforded by the BumSR TCS. Although species that compose the gut microbiota may vary between the avian and human host, the products of metabolism by the lower intestinal microbiota of each are similar. Thus, *C. jejuni* can employ the BumSR TCS to detect identical products of metabolism by the microbiota in multiple hosts instead of relying on different sensing systems to be specialized in detecting an avian-specific factor for commensalism and a different human-specific factor for infection to lead to pathogenesis of diarrheal disease.

Ideally, we aim to study the ability of the BumSR TCS to detect its specific cues and possibly others in a natural host, such as during the colonization of chickens. This analysis would require using either a germ-free chicken host or one with a limited, defined microbiota. Then, a goal would be to manipulate or introduce a member of the microbiota to produce a metabolic cue for BumS. Alternatively, the cue itself may be introduced at the right physiological levels in the lower intestinal tract. This type of manipulation may allow us to determine how the concentration of one and only one specific cue impacts BumSR signal transduction required for colonization. However, there are multiple complications in performing this type of study due to our identification of multiple cues sensed by the BumSR TCS. First, chickens and other avian species are natural, ideal models for the colonization of *C. jejuni*. However, chicken models without a gut microbiota or with a defined microbiota are underdeveloped compared to murine models. Performing these types of studies with *C. jejuni* in murine systems is not ideal either as *C. jejuni* does not colonize healthy, immunocompetent germ-free mice or mice with a defined microbiota efficiently, and colonization does not occur across an extended period of time. In addition, if a model was used that involved altering the microbiota, experimentation would require that the microbiota be manipulated in such a way so that only one cue for BumS—butyrate, isobutyrate, or isovalerate—would be produced or altered to fully reveal the impact on how changing levels of a metabolic cue impacts the ability of the BumSR TCS to sense and respond to that cue. Currently, it is unknown in many of these model systems what member(s) of the microbiota are responsible for the fermentation of branched-chain amino acids to produce isobutyrate or isovalerate. Considering these hurdles, we have already shown the importance of the BumSR TCS in colonization of the natural avian host and infection of humans for diarrheal disease (12, 23). These defects in the infection of hosts are presumably due to the inability of *bumSR* mutants to efficiently detect butyrate, isobutyrate, and isovalerate as landmark lower intestinal cues and alter gene expression appropriate for infection.

We originally discovered BumSR as the first bacterial TCS to our knowledge to employ a sensor that strictly functions as a phosphatase for signal transduction to mediate a response. However, it was unknown how the detection of cues mechanistically impacts phosphatase activity for these types of sensors and many other canonical TCS HKs. Identification of isobutyrate and isovalerate as direct cues detected by BumS enabled us to pursue this line of investigation. Both exogenous isobutyrate and isovalerate were detected by *C. jejuni* through the BumSR TCS and inhibited the *in vitro* phosphatase activity of BumS for P-BumR at a relevant physiological concentration found in the lower intestines of hosts. Through circular dichroism, we not only provided further evidence for isobutyrate and isovalerate as direct cues for BumS but also developed a hypothesis for how these cues exert their effects on BumS. Binding isobutyrate or isovalerate apparently caused a restructuring of BumS and reduced thermostability, which likely caused the reduction in phosphatase activity we observed in our assays. Disrupting tertiary protein structure upon binding cues could be a convenient strategy to inhibit a sensor like BumS that only possesses a sole enzymatic activity. In this case, inactivating BumS by this mechanism does not require maintaining a particular protein conformation to preserve kinase activity, which BumS lacks. The situation is likely different for many canonical TCS sensors in which the detection of cues across a range of concentrations causes toggling between their opposing kinase and phosphatase states (8, 9). A reorganization of tertiary structure upon binding cues by canonical TCS sensors may be detrimental to both their enzymatic activities and limit, or potentially destroy, their ability to promote signal transduction.

An interesting question arises from our work for how a sensor in a bacterial cell specifically binds and detects multiple intestinal metabolites. Due to BumS being predicted as a cytoplasmic sensor, we suspect a permease would be necessary for the transport of its cues into the cytoplasm for detection by the BumS PAS domain. However, we have yet to identify a transporter for butyrate, isobutyrate, or isovalerate. PAS domains are common sensory modules across many domains of life (46), but no PAS domain has been associated with the detection of isobutyrate or isovalerate directly or butyrate indirectly. Furthermore, no TCS in bacteria has been identified to detect these metabolites prior to this study. Through bioinformatic analysis, we found that the BumS PAS domain belongs to cluster 14 of the PAS domain family. Members of this domain were previously shown to utilize a conserved tryptophan residue along with other predicted residues to bind FAD as a co-factor for redox sensing. The PAS domain of BumS lacks this conserved tryptophan and has replaced it with a leucine (L68) while conserving some of these other residues. Our mutational analysis of the BumS PAS domain revealed that alteration of at least two conserved residues L68 and N83 alone or together in the BumS_QUAD_ variant caused a severe reduction in the detection of exogenous isobutyrate, isovalerate, and butyrate. However, alteration of these residues together caused BumS to be significantly blind, but not completely blind, to cues. These findings suggest more residues are involved in sensing these metabolites.

Regardless, an impaired ability to sense BSCFAs had a detrimental effect on colonization in chicks. Deleting *bumS* did not greatly hinder colonization of chicks, suggesting that maximal levels of P-BumR to highly repress transcription of certain genes like *peb3* and highly activate expression of others like *Cjj0438* within the BumSR regulon does not severely impact colonization, at least up to day 7 post-infection. However, having a partially blind BumS appears to cause a lack of control of its phosphatase activity, leading to some intermediate level of P-BumR in the bacterium during *in vivo* growth. We suspect that the level of expression of the BumSR regulon achieved by this intermediate level of P-BumR is not ideal and remains relatively static, causing lower colonization by *C. jejuni*. To corroborate the apparent reduced ability of BumS_QUAD_ to sense these exogenous BSCFAs *in vivo*, the *in vitro* phosphatase activity of BumS_QUAD_, unlike WT BumS, was largely unaffected by isobutyrate or isovalerate. We previously found that *C. jejuni* Δ*bumR* (lacking any P-BumR) had a significant colonization defect (12). It would be interesting to compare the ability of Δ*bumS*, Δ*bumR*, or *bumS*_QUAD_mutants to persist for multiple weeks in the colonization tract of chicks.

One limitation we encountered in our studies was the dynamic range of metabolites that we had to use in the phosphatase assays and circular dichroism analysis to detect an impact on BumS. As we showed in this work, BumS in *C. jejuni* detected all three of its cues when they were present at physiological concentrations, which are naturally in the μm to mM range in hosts. This range of cues is relatively higher than the concentration of cues detected by many other TCS sensors. We suspect that BumS has evolved to detect its cues at these concentrations in hosts to accurately control its phosphatase activity to promote the correct transcriptional response conducive to colonization and infection. This natural detection of its cues at low mM concentration caused recombinant BumS to be refractory for many biophysical assays like isothermal titration calorimetry, microscale thermophoresis, or surface plasmon resonance to monitor *in vitro* receptor-ligand interactions. These techniques are ideal for receptors that are more sensitive in detection of their cues at much lower levels (e.g., nM to μM range). These complications combined also precluded us from *in vitro* analyses of single BumS point mutants such as BumS_L68A_ and BumS_N83A_. Therefore, we could not characterize the impact of these residues on the recognition of cues by BumS and its phosphatase activity, although these residues significantly impacted BumS to sense its cues when the respective BumS mutants were expressed in the *C. jejuni* cell. These same hurdles also prevented us from performing a fine analysis of the thermostability of BumS_QUAD_ upon binding isobutyrate or isovalerate. Due to its reduced sensitivity in detecting these cues and its retention of phosphatase activity in the presence of these cues, we suspect that fewer structural changes would occur to BumS_QUAD_ in the presence of lower concentrations of these metabolites.

A major question that remains unanswered from this work and our previous work is how butyrate is sensed by BumS. The BumSR TCS is required to sense exogenous butyrate, but this metabolite did not inhibit the *in vitro* phosphatase activity of BumS or alter thermostability as analyzed by circular dichroism. Our current hypothesis is that butyrate may be converted by *C. jejuni* into a cue or cause the production of a cue by *C. jejuni* that is directly detected by BumS. If so, this unknown product may possess similar features as isobutyrate and isovalerate since L68 and N83 of the BumS PAS domain were required to efficiently detect all three metabolites. In nature, there are no known enzymes or pathways for the conversion of butyrate to isobutyrate or isovalerate. Additionally, *C. jejuni* lacks known genes for catabolism of butyrate, isobutyrate, or isovalerate. However, we cannot exclude the possibility that *C. jejuni* encodes unidentified factors for these processes. Future work will involve designing specific screens or selections to identify genes of *C. jejuni* that are required for the indirect sensing of butyrate.

Our work has not only expanded the repertoire of cues detected by the BumS TCS of *C. jejuni* that is required for efficient colonization of avian and human hosts but also identified isobutyrate and isovalerate as intestinal metabolites that are direct cues for BumS. Finding three specific cues for a single TCS in the natural niche of multiple hosts is an important discovery for understanding how this bacterium can colonize multiple avian and animal hosts in nature and agriculture while also promoting infection of humans to contribute to one of the leading causes of bacterial diarrheal disease throughout the world. We also revealed how these cues structurally alter BumS to impact its phosphatase activity to modulate signal transduction. Many interesting questions remain to be explored for how this sensor phosphatase-driven TCS mechanistically functions to alter the transcriptome of *C. jejuni* for association with hosts. One question involves the consequence of employing a sensor that only functions as a phosphatase. Since BumS does not function as an autokinase to facilitate phosphotransfer to BumR to produce P-BumR, a non-cognate phosphodonor must exist in the *C. jejuni* cell for BumR. We are currently pursuing factors and pathways that could provide a second signaling input to the BumSR TCS by contributing phosphodonors for BumR and how BumS controls levels of P-BumR pools in response to exogenous intestinal metabolites.

## MATERIALS AND METHODS

### Bacterial strains, plasmids, and growth

*C. jejuni* strains were routinely grown from freezer stocks in microaerobic conditions (10% CO_2_, 5% O_2_, and 85% N_2_) created by a tri-gas incubator on Mueller-Hinton (MH) agar containing 10 µg/mL trimethoprim at 37°C for 48 h. Strains were then restreaked onto MH agar with trimethoprim, unless otherwise stated, and grown for an additional 16 h. Agar was added to MH broth to 1.7% (wt/vol) to create MH agar. Antibiotics were added to the media when needed at the following concentrations: 15 µg/mL chloramphenicol, 100 µg/mL kanamycin, or 0.1, 0.5, 1, or 2 mg/mL streptomycin. When noted, strains were grown on *Campylobacter* defined medium (CDM), which contains all 20 amino acids, specific keto acids, and other nutrients at concentrations to support growth (47). CDM was solidified by adding agar to 1.7% (wt/vol). *E. coli* DH5α, BL21 (DE3), and TOP10 strains were grown on LB (Lennox L) agar or LB broth containing 100 µg/mL ampicillin, 100 µg/mL kanamycin, or 15 µg/mL chloramphenicol when necessary. *C. jejuni* strains were stored at −80°C in a mixture of 85% MH broth and 15% glycerol. *E. coli* strains were stored at −80°C in a mixture of 80% LB broth and 20% glycerol.

### Construction of *C. jejuni* mutants

All bacterial strains and plasmids constructed and used in experiments are listed in Tables S2 and S3. *C. jejuni* mutants were constructed with plasmids purified from *E. coli* that were introduced into *C. jejuni* by electroporation (39).

For creation of the BumS point mutants, SmaI-digested BumS point mutants, SmaI-digested *cat-rpsL* antibiotic cassettes from pDRH265 were first inserted into HapI site of *bumS* to create pPML107. This plasmid was then electroporated into DRH461 (81–176 *rpsL*^Sm^Δ*astA*) (39). Transformants were recovered on MH agar plates containing chloramphenicol, and mutations were verified by colony PCR to result in NR576 (81–176 *rpsL*^Sm^Δ*astA bumS::cat-rpsL*). Primers containing desired point mutations were used to amplify *bumS* from DRH212 (81–176 *rpsL*^Sm^) with 750 bases upstream and downstream of the gene. PCR fragments were inserted into the EcoRI sites of pUC19 via Gibson Assembly (New England Biolabs) to result in pNR560 (pUC19::*bumS*_S20A_), pNR559 (pUC19::*bumS*_H51A_), pNR556 (pUC19::*bumS*_L68A_), and pNR557 (pUC19::*bumS_N83A_*). A gene block encoding all four mutations (S20A, H51A, L68A, and N83A; Integrated DNA Technologies) was used as a template for PCR with primers that amplified the mutant locus prior to insertion into the EcoRI sites of pUC19 by Gibson Assembly to result in pNR654 (pUC19::*bumS*_QUAD_). These plasmids were then electroporated into NR576 to replace *bumS::cat-rpsL* with each *bumS* mutant allele. All transformants were recovered on MH agar containing 0.1 to 2 mg/mL streptomycin. Streptomycin-resistant, chloramphenicol-sensitive transformants were screened by colony PCR and sequenced to confirm correct construction of the mutants to result in NR621 (81–176 *rpsL*^Sm^Δ*astA bumS*_S20A_), NR619 (81–176 *rpsL*^Sm^Δ*astA bumS*_H51A_), NR608 (81–176 *rpsL*^Sm^Δ*astA bumS*_L68A_), NR566 (81–176 *rpsL*^Sm^Δ*astA bumS*_N83A_), and NR663 (81–176 *rpsL*^Sm^Δ*astA bumS*_QUAD_).

### Arylsulfatase transcriptional reporter assays

For creation of the *peb3::astA* transcriptional reporter in *C. jejuni* strains, pPML873 (19) was electroporated into PML363 (81–176 *rpsL*^Sm^Δ*astA* Δ*bumS*), NR621 (81–176 *rpsL*^Sm^Δ*astA bumS*_S20A_), NR619 (81–176 *rpsL*^Sm^Δ*astA bumS*_H51A_), NR602 (81–176 *rpsL*^Sm^Δ*astA bumS*_L68A_), NR566 (81–176 *rpsL*^Sm^Δ*astA bumS*_N83A_), and NR663 (81–176 *rpsL*^Sm^Δ*astA bumS*_QUAD_). Transformants were selected on MH agar with kanamycin and screened by colony PCR for verification to result in the recovery of PML912 (81–176 *rpsL*^Sm^Δ*astA*Δ*bumS peb3::astA-kan*), NR624 (81–176 *rpsL*^Sm^Δ*astA bumS*_S20A_ *peb3::astA-kan*), NR622 (81–176 *rpsL*^Sm^Δ*astA bumS*_H51A_*peb3::astA-kan*), NR612 (81–176 *rpsL*^Sm^Δ*astA bumS*_L68A_ *peb3::astA-kan*), NR604 (81–176 *rpsL*^Sm^Δ*astA bumS*_N83A_ *peb3::astA-kan*), and NR669 (81–176 *rpsL*^Sm^Δ*astA bumS*_QUAD_ *peb3::astA-kan*).

For the generation of *Cjj0438::astA* transcriptional reporter strains, DRH461, PML363, and PML337 were electroporated with pDRH3457 to create PML718 (81–176 *rpsL*^Sm^Δ*astA Cjj0438::astA-kan*), PML721 (81–176 *rpsL*^Sm^Δ*astA*Δ*bumR Cjj0438::astA-kan*), and PML736 (81–176 *rpsL*^Sm^Δ*astA*Δ*bumS Cjj0438::astA-kan*).

Arylsulfatase assays were performed to measure the level of transcription of *peb3::astA* or *Cjj0438::astA* on the chromosome of *C. jejuni* Δ*astA* strains as previously described (48–50). Strains for arylsulfatase assays were first grown from freezer stocks, and then, each strain was restreaked on MH containing up to 12.5 mM butyrate, isobutyrate, and/or isovalerate and grown for 16 h at 37°C in microaerobic conditions. Arylsulfatase assays were performed with each strain in triplicate. The level of *peb3::astA* or *Cjj0438::astA* expression in each strain was calculated relative to the expression in WT *C. jejuni* Δ*astA* strain or the relevant mutant strain grown in MH in the absence of metabolite, which was set to 100 units.

### Semi-quantitative real-time-PCR (qRT-PCR) analysis

After the growth of *C. jejuni* strains from freezer stocks on MH agar with 10 µg/mL trimethoprim, strains were restreaked onto MH agar and grown for an additional 16 h. *C. jejuni* growth was then suspended from the plates in PBS and diluted into 25 mL of CDM alone or CDM with 0.25 to 12.5 mM butyrate, isobutyrate, or isovalerate. Strains were grown statically at 37°C in microaerobic conditions for 8 h to achieve mid-log phase growth. Total RNA was extracted with TRIzol (Ambion) and RNA was treated with DNaseI (Invitrogen). RNA was diluted to a concentration of 5 ng/µL before analysis. A one-step qRT-PCR was performed using MultiScribe Reverse Transcriptase (Invitrogen) and PowerTrack SYBR Green Master Mix (Applied Biosystems) with the QuantStudio 3 system (Applied Biosystems) following the ΔΔCt method. *secD* mRNA detection was used as an endogenous control since transcript levels were consistent across strains and conditions, and similar to expression levels of target genes. mRNA transcript levels in DRH212 grown in CDM alone served as WT controls to determine the relative gene expression.

### Expression and purification of recombinant proteins

Purification of glutathione S-transferase (GST)-BumR recombinant proteins were purified as previously described with slight modifications (19). Briefly, *E. coli* BL21 (DE3) was transformed with pPML165 and then grown in 2xYT medium to mid-log phase prior to induction with 300 µM IPTG. GST-BumR protein was purified from the soluble fraction with glutathione Sepharose beads (GE Healthcare). Following cleavage of the GST tag by thrombin and removal of thrombin by benzamidine Sepharose following the manufacturer’s instructions (GE Healthcare), recombinant BumR was recovered. Glycerol was added to a final concentration of 10%, and proteins were stored at −80°C.

A plasmid to express WT BumS-Myc-6XHis was constructed with primers to amplify WT *bumS* from the second to penultimate codon from *C. jejuni* 81–176 genomic DNA. This PCR fragment was inserted into NcoI and HinDIII-digested pBAD/Myc-HisA via Gibson Assembly to generate pNR251 (pBAD/Myc-HisA::*bumS*) and then transformed into TOP10. This method was also used to generate pNR656 (pBAD/Myc-HisA::*bumS*_QUAD_) except the template for PCR amplification was pNR654 (pUC19::*bumS*_QUAD_). Plasmids were verified for correct construction by sequencing.

For the purification of WT BumS-Myc-6XHis and BumS_QUAD_-Myc-6XHis, 0.5 mL of overnight cultures of *E. coli* TOP10/pNR251 or TOP10/pNR656 was inoculated into 20 mL of 2xYT media with 100 µg/mL ampicillin. Cultures were grown at 37°C under shaking conditions until growth reached an OD_600_ of 0.5–0.9. Cultures were then diluted 1:50 into 500 mL of 2xYT with 100 µg/mL ampicillin and grown until OD_600_ of 0.8–0.9. Cultures were then induced with arabinose at a final concentration of 0.02% overnight at 16°C with shaking. Bacterial cells were collected by centrifugation and then suspended in 50 mL of 50 mM NaH_2_PO_4_, 300 mM NaCl, and 10 mM imidazole. Cells were passed through an EmulsiFlex-C5 disruptor at 15,000–20,000 lb/in^2^ to lyse cells. The soluble fraction was recovered by centrifugation at 20,000 × *g* for 1 h. Recombinant protein was purified from the soluble fraction via fast protein liquid chromatography (FPLC) using an NGC chromatography system and EconoFit Profinity IMAC Ni-charged column (Bio-Rad). Proteins were eluted with a 0%–100% gradient of 250 mM imidazole. The eluted protein was then loaded onto a Bio-Gel P-6 Desalting column (Bio-Rad) for buffer exchange with 10 mM HEPES and 300 mM NaSO_4_. Glycerol was added to the final preparation at a final concentration of 10% prior to storage at −80°C.

### *In vitro* BumS phosphatase assays for P-BumR

Radiolabeled Ac[^32^P] was generated as previously described with some modifications (51). For BumR phosphorylation, 0.3 units of *E. coli* acetate kinase (AckA; Sigma-Aldrich) were incubated in buffer containing 50 mM Tris HCl pH 7.6, 120 mM potassium acetate, 20 mM magnesium chloride, 1 mM DTT, 0.5 mM ATP, 5 µCi ATP[γ−32P], and 10% glycerol in a total volume of 7.5 µL for 2 h at room temperature. Recombinant BumR (20 pmol) was then added to achieve a total volume of 10 µL. Phosphorylation of BumR was allowed to occur for 20 min at 37°C. Recombinant WT BumS-Myc-6XHis or BumS_QUAD_-Myc-6XHis were added to the reaction to create a 1:2 BumS:BumR ratio in a total volume of 20 µL. When noted, butyrate, isobutyrate, isovalerate, 4-methy-2-oxovalerate, 3-methyl-2-oxobutyrate, and/or 3-methyl-2-oxopentanoate were added up to 12.5 mM to BumS proteins prior to their addition to P-BumR. Phosphatase reactions were allowed to proceed for 5 min at 37°C. Reactions were terminated with 2X Laemmli buffer containing 5% BME, and proteins were separated by 4%–20% TGX gradient gels (Bio-Rad) without prior boiling of samples. Gels were dried and then analyzed with a Typhoon FLA 9500 phosphorimager according to the manufacturer’s instructions (Amersham Biosciences). All densitometry calculations were done using Bio-Rad Image Lab software. For calculation of BumS phosphatase activity, the amount of signal for P-BumR incubated with WT BumS alone was subtracted from the amount of signal for P-BumR alone. This difference was then set as 100% phosphatase activity for WT BumS in the absence of metabolites. The amount of phosphatase activity for WT BumS mixed with metabolites is reported relative to WT BumS in the absence of metabolites. Similarly, the level of phosphatase activity of BumS_QUAD_ with metabolites is reported relative to BumS_QUAD_ in the absence of metabolites. All assays were performed in triplicate to derive a mean phosphatase activity with standard deviation.

### BumS thermostability assays

Thermostability analysis of BumS in the presence and absence of metabolites by circular dichroism spectroscopy was performed using the Jasco J-815 spectrometer and a cuvette with a 1.0 mm path length. Each sample contained a final concentration of 0.2 mg/mL of recombinant WT BumS-Myc-6XHis in 10 mM HEPES, 300 mM NaSO4, and 10% (vol/vol) glycerol. When needed, isobutyrate, isovalerate, or butyrate was added to a final concentration from 12.5 to 50 mM. Spectral readings were recorded at 222 nm from 25°C to 95°C (298–368K) every 1°C; the temperature was held at the target for at least 1 min prior to data acquisition. A data-integration time of 4 s was used. Temperature was modulated by the Jasco Peltier temperature control system. The melting data (*CD(T*)) were fitted to a standard logistic-type curve using a custom Python script:


$$CD\left(T\right)=\frac{\left(b_{1}+m_{1}T\right)+\left(b_{2}+m_{2}T\right)e^{-\Delta H\left(1-T/T_{m\text{,app}}\right)/RT}}{1+e^{-\Delta H\left(1-T/T_{m\text{,app}}\right)/RT}},$$

where *b*_1_ and *m*_1_ are intercept and slope parameters for the left portion of the curve, respectively; *b*_2_ and *m*_2_ are intercept and slope parameters for the right side of the curve, respectively; *T_m_*_, app_ is the apparent melting temperature (in K); and *R* is the universal gas constant. Apparent melting temperature is reported here because equation (1) assumes the reversibility of the folding, but this was not tested.

### Identifying potential binding sites within the BumS PAS domain *in silico*

The PAS domain of *C. jejuni* BumS (NCBI accession: EAQ72684.1/1–121 or WP_153887626.1/1–121) was used as a query in Basic Local Alignment Search Tool (BLAST) searches against sequences from the previously defined PAS domain classification system (34). PAS domain sequences in cluster 14 were identified as homologs of the BumS PAS domain. Key residues were identified based on our previous analysis of cluster 14 (34). Multiple sequence alignments were built using MAFFT (52). Sequence logos were generated using WebLogo3 (53). Structures of PAS domains from BumS and CetB (cluster 14) were modeled using AlphaFold (54) and visualized using PyMOL (55). The BumS PAS domain was then used in a BLAST search against the NCBI Reference Sequence protein database (RefSeq) with default parameters (56). Several PAS domain homologs from different species of the order *Campylobacterales* were selected from the BLAST result for comparative analysis (NCBI accessions: WP_153887626.1, WP_131952296.1, WP_148563445.1, WP_142692976.1, WP_193151068.1, WP_151901227.1, WP_013326056.1, WP_129094579.1, WP_129012495.1, WP_069478740.1, and WP_115428278.1).

### Immunoblotting analysis of *C. jejuni* proteins

After growing *C. jejuni* strains from frozen stocks, strains were restreaked on MH with kanamycin and grown for 16 h at 37°C in microaerobic conditions. Cells were resuspended in PBS and diluted to OD_600_ 0.8. For whole cell lysates, 1 mL of samples was centrifuged and washed once with 1 mL of PBS. Pellets were resuspended in 50 µL of 1× Laemmli buffer with 5% BME and boiled for 10 min. Whole-cell lysate samples (30 µL) were separated on a 4%–20% TGX gradient gels (Bio-Rad) for the detection of BumS, and 12.5 µL of the sample was separated similarly for the detection of RpoA as a control. Proteins were detected with dilutions of specific M226 murine antisera for BumS (19) and specific GP275 guinea pig antisera for RpoA (57) by applying the antisera to membranes for 1 or 2 h, respectively. Appropriate horseradish peroxidase-conjugated goat antibodies were used as secondary antibodies to develop immunoblots. Immunoblots were developed with a Western Lightning Plus ECL kit (Revvity) and imaged using the Bio-Rad ChemiDoc system.

#### Chick colonization assays

The ability of WT *C. jejuni* and isogenic mutants to colonize chicks after oral inoculation was determined as previously described (44). Briefly, fertilized chicken eggs (SPAFAS) were incubated for 21 days at 37.5°C with appropriate humidity and rotation in a Digital Sportsman model 1502 incubator (Georgia Quail Farms Manufacturing Company). One day after hatch, chicks were orally inoculated with 100 µL of phosphate-buffered saline (PBS) containing approximately 10–200 CFU WT or mutant strains. Strains were prepared for infection after 16 h growth at 37°C under microaerobic conditions on MH agar by suspending *C. jejuni* strains in MH broth. Dilution series in PBS were performed to achieve the appropriate inoculum for oral gavage of chicks. Dilutions of the inoculum were plated on MH agar to assess the number of bacteria in each inoculum. At day 7 post-infection, the chicks were sacrificed. The proximal small intestine, distal small intestine, cecal, or large intestine contents were removed and suspended in PBS and serial dilutions were plated on MH agar containing trimethoprim and cefoperazone. Following 72 h of growth at 37°C in microaerobic conditions, bacteria were counted to determine CFU per gram of organ content. Recovered colonies were analyzed by colony PCR to verify that WT and mutant strains were isolated from infected chicks, respectively.

## Data Availability

All data, strains, and plasmids are available upon request. All data acquired during this study are included in the article and/or supplemental material.

## SUPPLEMENTAL MATERIAL

Supplemental Material mbio.03278-24-s0001.docx Figures S1-S3 and Tables S2 and S3.

Table S1 mbio.03278-24-s0002.xlsx PAS domains homologous to the *C. jejuni* BumS PAS domain.

ASM does not own the copyrights to Supplemental Material that may be linked to, or accessed through, an article. The authors have granted ASM a non-exclusive, world-wide license to publish the Supplemental Material files. Please contact the corresponding author directly for reuse.
