# Supplementary material for: The Campylobacter jejuni BumS sensor phosphatase detects the branched short-chain fatty acids isobutyrate and isovalerate as direct cues for signal transduction
Source: mBio. 2024 Dec 13;16(2):e03278-24. doi: 10.1128/mbio.03278-24 (PMC11796366; doi:10.1128/mbio.03278-24)
Supplement: Supplemental Material — Figures S1-S3 and Tables S2 and S3. [file mbio.03278-24-s0001.docx]

**Figure S1. BumS phosphatase activity for P-BumR in the presence of isobutyrate, isovalerate, or both.** A representative assay of BumS phosphatase activity for ^32^P-BumR in the presence of isobutyrate (IB) and isovalerate (IV) that was performed in triplicate to derive the quantitative BumS phosphatase activity shown in Figure 4C. The indicated concentration of metabolites was added to BumS before addition of ^32^P-BumR.

**
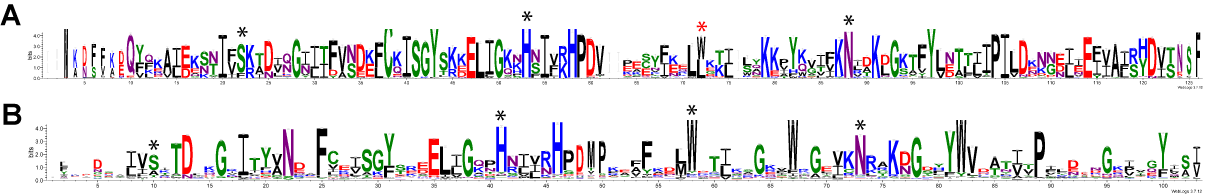
**

**Figure S2.** **Comparison of PAS domains from Cluster 14 and BumS homologs.**

Sequence logos of (A) PAS domains most homologous to that of BumS and (B) PAS domains of proteins from cluster 14. Key residues that may be involved in ligand sensing were labeled by asterisks. Some PAS domains homologous to that of BumS have the W to L substitution (red asterisk).

**Figure S3. Comparison of WT BumS and BumS_QUAD_ phosphatase activity for P-BumR in the presence of isobutyrate or isovalerate.** A representative assay of BumS phosphatase activity for ^32^P-BumR in the presence of isobutyrate (IB) or isovalerate (IV) that was performed in triplicate to derive the quantitative BumS phosphatase activity shown in Figure 7. The indicated concentration of metabolites were added to BumS before addition of ^32^P-BumR.

**Table S2. Bacterial strains used in this study.**

| **Strain** | **Genotype** | **Source/Reference** |
| --- | --- | --- |
| ***E. coli* strains** | | |
| DH5α | *supE44* Δ*lacU169* (ϕ80*lacZ*ΔM15) *hsdR17* *recA1* *endA1* *gyrA96 thi-1 relA1* | Invitrogen |
| BL21 (DE3) | *fhuA2 [lon] ompTgal (λ DE3) [dcm]* Δ*hsdS λDE3 = λ sBamHIo* Δ*EcoRI-B int∷(lacI∷PlacUV5∷T7 gene1) i21* Δ*nin5* | New England Biolabs |
| TOP10 | *F–mcrA* Δ*(mrr-hsdRMS-mcrBC) φ80lacZΔM15* Δ*lacX74 recA1 araD139* Δ*(ara-leu)7697 galU galK λ–rpsL^(StrR)^ endA1 nupG* | Invitrogen |
|  | | |
| ***Campylobacter jejuni* strains** | | |
|  |  |  |
| DRH212 | 81–176 *rpsL*^Sm^ | (1) |
| DRH461 | 81–176 *rpsL*^Sm^ Δ*astA* | (2) |
| PML337 | 81–176 *rpsL*^Sm^ Δ*astA* Δ*bumR* | (3) |
| PML363 | 81–176 *rpsL*^Sm^ Δ*astA* Δ*bumS* | (4) |
| PML718 | 81–176 *rpsL*^Sm^ Δ*astA Cjj0438*::*astA-kan* | This study |
| PML721 | 81–176 *rpsL*^Sm^ Δ*astA* Δ*bumR Cjj0438*::*astA-kan* | This study |
| PML736 | 81–176 *rpsL*^Sm^ Δ*astA* Δ*bumS Cjj0438*::*astA-kan* | This study |
| PML908 | 81–176 *rpsL*^Sm^ Δ*astA* Δ*bumR peb3*::*astA-kan* | (4) |
| PML912 | 81–176 *rpsL*^Sm^ Δ*astA* Δ*bumS peb3*::*astA-kan* | This study |
| PML921 | 81–176 *rpsL*^Sm^ Δ*astA peb3*::*astA-kan* | (4) |
| NR566 | 81–176 *rpsL*^Sm^ Δ*astA bumS*_N83A_ | This study |
| NR576 | 81-176 Δ*astA bumS*::*cat-rpsL* | This study |
| NR604 | 81–176 *rpsL*^Sm^ Δ*astA bumS*_N83A_ *peb3*::*astA-kan* | This study |
| NR608 | 81–176 *rpsL*^Sm^ Δ*astA bumS*_L68A_ | This study |
| NR612 | 81–176 *rpsL*^Sm^ Δ*astA bumS*_L68A_ *peb3*::*astA-kan* | This study |
| NR619 | 81–176 *rpsL*^Sm^ Δ*astA bumS*_H51A_ | This study |
| NR621 | 81–176 *rpsL*^Sm^ Δ*astA bumS*_S20A_ | This study |
| NR622 | 81–176 *rpsL*^Sm^ Δ*astA bumS*_H51A_ *peb3*::*astA-kan* | This study |
| NR624 | 81–176 *rpsL*^Sm^ Δ*astA bumS*_S20A_ *peb3*::*astA-kan* | This study |
| NR663 | 81–176 *rpsL*^Sm^ Δ*astA bumS*_QUAD_ | This study |
| NR669 | 81–176 *rpsL*^Sm^ Δ*astA bumS*_QUAD_ *peb3*::*astA-kan* | This study |

| **Plasmid** | **Genotype/Description** | **Source/Reference** |
| --- | --- | --- |
| pUC19 | Amp^R^ | New England Biolabs |
| pBAD/Myc-HisA | Amp^R;^ used for the expression of C-terminal Myc-6XHis tagged proteins | Invitrogen |
| pDRH3457 | pUC19 with the *Cjj0438* promoter from 81-176 fused to *astA-kan* | This study |
| pPML107 | pUC19 with *bumS::cat-rpsL* cloned into the BamHI site | This study |
| pPML165 | pGEX-4T-2 with *BumR* coding sequence from codon 2 to the stop codon cloned into BamHI site | (3) |
| pPML873 | pUC19 with the *peb3* promoter from 81-176 fused to *astA-kan* | (4) |
| pNR251 | pBad/Myc-HisA with *bumS* from codon two to penultimate codon cloned into the NcoI and HinDIII sites | This study |
| pNR556 | pUC19 with *bumS_L68A_* including 750 bp upstream and downstream cloned into the EcoRI site | This study |
| pNR557 | pUC19 with *bumS_N83A_* including 750 bp upstream and downstream cloned into the EcoRI site | This study |
| pNR559 | pUC19 with *bumS_H51A_* including 750 bp upstream and downstream cloned into the EcoRI site | This study |
| pNR560 | pUC19 with *bumS_S20A_* including 750 bp upstream and downstream cloned into the EcoRI site | This study |
| pNR654 | pUC19 with *bumS*_QUAD_ including 750bp upstream and downstream cloned into the EcoRI site | This study |
| pNR656 | pBad/Myc-HisA with *bumS*_QUAD_ from codon two to penultimate codon cloned into the NcoI and HinDIII sites | This study |

**Table S3. Plasmids used in this study.**

**References**

1. **Hendrixson DR, Akerley BJ, DiRita VJ.** 2001. Transposon mutagenesis of *Campylobacter jejuni* identifies a bipartite energy taxis system required for motility. Mol Microbiol **40:**214-224.

2. **Hendrixson DR, DiRita VJ.** 2003. Transcription of σ^54^-dependent but not σ^28^-dependent flagellar genes in *Campylobacter jejuni* is associated with formation of the flagellar secretory apparatus. Mol Microbiol **50:**687-702.

3. **Luethy PM, Huynh S, Parker CT, Hendrixson DR.** 2015. Analysis of the activity and regulon of the two-component regulatory system composed by Cjj81176_1484 and Cjj81176_1483 of *Campylobacter jejuni*. J Bacteriol **197:**1592-1605.

4. **Goodman KN, Powers MJ, Crofts AA, Trent MS, Hendrixson DR.** 2020. *Campylobacter jejuni* BumSR directs a response to butyrate via sensor phosphatase activity to impact transcription and colonization. Proc Natl Acad Sci U S A **117:**11715-11726.
